# The Impact of an Electronic Prescribing Template with Decision Support upon the Prescribing of Subcutaneous Infusions at the End of Life in a Community Setting: A Future Vision for Community Palliative Care

**DOI:** 10.3390/pharmacy10050112

**Published:** 2022-09-09

**Authors:** Y. K. Au, L. Baker, J. Hindmarsh

**Affiliations:** 1 South Tyneside and Sunderland NHS Foundation Trust, Sunderland SR4 7TP, UK; 2 St. Benedict’s Hospice, Specialist Centre for Palliative Care, Sunderland SR2 0NY, UK; 3 Advanced Clinical Pharmacist, South Tyneside and Sunderland Foundation Trust, Department of Pharmacy, Sunderland Royal Hospital, Kayll Road, Sunderland SR4 7TP, UK

**Keywords:** palliative care, electronic prescribing, subcutaneous infusions, community care, pharmacy practice

## Abstract

**Objectives**: To assess the impact of an electronic prescribing template with decision support upon the frequency of prescription errors, guideline adherence (relating to dose ranges), and prescription legality when prescribing continuous subcutaneous infusions (CSCI) in a palliative demographic. **Design, setting, and participants:** Before-and-after study across a large UK city utilizing local prescribing data taken from patients receiving end-of-life care. **Intervention:** An electronic prescribing template with decision support. **Main outcome measures:** The following were assessed: (1) the rate of prescription errors; (2) the proportion of prescriptions specifying a dose range and if the specified range complied with local recommendations; and (3) the proportion of prescriptions specifying legal mixing directions. **Results:** The intervention was associated with a significant reduction in errors of omission, with all prescriptions clearly stating drug indication, route of administration, drug dose, and infusion duration. The numbers of continuous subcutaneous infusion prescriptions that specified dose ranges were similar at baseline and post-intervention, at 71% (*n* = 122) and 72% (*n* = 179), respectively. At baseline, 69% (*n* = 84) of CSCI prescriptions specifying a dose range were deemed safe, and post-intervention, 97% (*n* = 173) were determined to be safe. At baseline, mixing directions were not specified correctly on any continuous subcutaneous infusion prescriptions, while post-intervention, such directions were correct on 75% (*n* = 157; *p* < 0.05) of the prescriptions. **Conclusions:** The intervention eliminated errors of omission, ensured the safety of prescribed dose ranges, and improved compliance with legislation surrounding the mixing of multicomponent infusions. Overall, the intervention has the potential to improve patient safety at the end of life and to increase the efficiency of community services.

## 1. Introduction

Palliative care is a complex clinical area, in which patients require highly individualized pharmacological management [1,2,3]. Symptoms experienced by the palliative demographic may increase in the last days to weeks of life and necessitate a combination of different pharmacological interventions to relive suffering [4,5]. Typical symptoms occurring at the end of life include pain, nausea, vomiting, agitation, and respiratory secretions. [1,2,3,4,5,6,7]. Although the preference is to administer medicines via the oral route, this is frequently not possible towards the end of life, as dysphagia, nausea, vomiting, bowel obstruction, and reduced consciousness frequently preclude oral administration and enteral absorption [1]. Consequently, in such circumstances, medicines are frequently administered via the subcutaneous route, as this provides a reliable route of administration that is much less invasive than intravenous or intramuscular injections [1,2,3,4,5,6].

In the UK, anticipatory medications (or just-in-case medicines) are frequently prescribed in anticipation of a patient becoming unwell [1,8]. Patients are typically prescribed an opioid (for management of pain and dyspnea), an antiemetic (for management of nausea and vomiting), an antisecretory agent (for management of respiratory secretions), and a benzodiazepine (for management of distress, anxiety, and agitation) [1,2,3,4,5,6,7,8]. Anticipatory medicines are routinely administered via the subcutaneous route and are widely prescribed across different healthcare sectors, including hospital-, hospice-, and community-based settings [1,8,9].

In a community setting, the prescribing of anticipatory medicines is encouraged to facilitate timely symptom control and prevent unnecessary hospital admissions [1,8,9]. Symptomatic patients are typically given an appropriate medicine as a bolus dose, which may subsequently be administered as a continuous subcutaneous infusion (CSCI) to provide around-the-clock symptom management [1,2,3,4,5,6,7,8]. Several studies [6,7,8] have also shown that the prescribing of anticipatory medicines in a community setting ensures prompt access to critical medications and enables rapid medicine administration and subsequent symptom control, especially in out-of-hours situations when medical support may be limited [8,9,10].

However, palliative prescribing in a community setting is also associated with potential risks. Faull et al. [11] demonstrated the different challenges faced by community health professionals when delivering palliative care, which included resourcing concerns and a lack of both professional and clinical experience in palliative care. Bowers et al. [8,12] also highlighted concerns surrounding patient safety, as there is a dearth of reliable data on how often anticipatory medicines are prescribed and subsequently used in community settings due to variation in palliative care practice.

Locally, practice-based reviews and incident reports highlight frequent errors relating to the prescribing and management of medicines at the end of life in community settings. Interestingly, a recent before-and-after study [13] undertaken in a large teaching hospital demonstrated that the adoption of electronically prescribed, mixed drug infusions was associated with a significant reduction in drug errors and improvements in service efficiency. Currently, there is a lack of research on how to improve prescribing practices within the community setting for palliative patients. As a result, we innovated an electronic drug chart with decision support to facilitate safe prescribing at the end of life in a community locality. To assess the impact of this intervention, an evaluation study with a before-and-after design was undertaken.

## 2. Materials and methods

### 2.1. Setting

The study was conducted across the entire community locality of a large UK city. Data were collected from across 39 general practitioner (GP) practices. At the time, CSCIs for palliative symptom management were prescribed by hand on paper drug charts.

### 2.2. Ethics Approvals

Both the local NHS Trust’s and the clinical commissioning group’s research and audit groups approved this study. As patient-identifiable data were not collected, ethical approval was not required.

### 2.3. Intervention

The intervention consisted of a new drug chart template with decision support, which was uploaded to EMIS health (at the time, this software was utilized by all GP practices across the city for clinical documentation and prescribing). The new drug chart template facilitated the prescribing of both anticipatory medicines and subcutaneous infusions and permitted drugs to be prescribed electronically and subsequently printed prior to placement in the patients’ homes.

The electronic decision support screened patient data contained within EMIS health (including previous prescriptions, past medical history, and biochemical parameters) and alerted the prescriber of relevant information to facilitate individualized prescribing. The main aspects included (1) identifying patients with severe renal dysfunction (eGFR < 30 mL/min/1.73 m^2^), which directed the prescriber to a drug chart where drug selection and doses were modified for renal impairment (for instance, recommending alfentanil in place of morphine for pain management); (2) highlighting existing oral therapy, which required conversion to a parenteral route of administration at the end of life (this included opioids, antiemetics, anticonvulsants, steroids, and antiparkinsonian medication); (3) recognizing contraindications between anticipatory medicines and pre-existing conditions (such as restricting the prescribing of antipsychotics in patients with Parkinson’s disease); and (4) providing physicochemical compatibility data for drug mixtures to be given by CSCI.

### 2.4. Study Design

A before-and-after methodology was used for this citywide study. Data were collected retrospectively from a defined six-month period before and a six-month period after the implementation of the new drug chart. Patients were included in the study if (1) they were prescribed a CSCI for end-of-life symptom management and (2) the infusion was administered.

### 2.5. Data Collection

The primary investigators (YKA and JH) produced a data collection tool that was disseminated to all primary care network pharmacists. Once a printed drug chart was no longer required, it was removed from the patient’s property by the district nursing team and stored locally in one of the primary care network hubs. From here, primary care network pharmacists reviewed the drug charts and completed the data collection tool, which was electronically sent to the primary investigators. Since patient-identifiable data were not recorded and the drug charts remained in their approved storage location, no additional permissions were required.

### 2.6. Prescription Errors

Each CSCI prescription was reviewed for omission errors. Such errors were defined as the erroneous exclusion of a drug’s name, dosage, diluent, dilution volume, date, signature, route of administration, or infusion length or rate.

All identified errors were examined and confirmed by a panel of two pharmacists in order to maintain uniformity in the prescription error determination process. Each eligible prescription was reviewed once, but if the prescription was later amended in any way (such as a change in dose), the superseding prescription was likewise evaluated. The study did not evaluate whether each prescription was clinically appropriate.

### 2.7. Dose Ranges

In instances where a dose range was prescribed, the appropriateness was assessed. A safe dose range was defined as one that permitted no more than a 50% increase in drug dose.

### 2.8. Mixing Directions

Appropriate mixing directions were defined as those which explicitly specified the discrete components of the infusion (for instance “morphine 10 mg to be mixed with midazolam 10 mg and diluted up to 23 mL with water for injection”). Ambiguous directions, such as “morphine can be mixed with any of the following” or “mix as per compatibility chart”, were deemed incorrect.

For patients prescribed two or more drugs to be administered via CSCI, the potential for the drugs to be mixed in a single infusion was determined using the syringe driver compatibility database available at palliativedrugs.com (accessed on 5 April 2022). This allowed the determination of how many syringe drivers required mixing directions versus how many actually contained such directions.

### 2.9. Sample Size and Statistical Analysis

We calculated that a minimum of 150 drug charts needed to be evaluated in each phase of the study to identify reductions in the outcomes. This estimate was based on early data collection and functionality testing, a power of 80%, and a significance threshold of 5%. A chi-squared test was used to compare nominal data and dichotomous outcomes (compliant or noncompliant). All statistical calculations were carried out using SPSS version 26, and *p* < 0.05 was regarded as significant.

## 3. Results

At baseline, data from 171 drug charts were collected from a total of 130 patients, and post-intervention, 250 drug charts from a total of 204 patients were assessed. The results are summarized in Table 1.

### 3.1. Prescriptions Errors

Drug names were clearly stated on 100% of all the CSCI prescriptions both pre- and post-investigation. At baseline, 64% (*n* = 110) of the drug charts stated the indication of each drug contained within the CSCI, whilst post-intervention, 100% (*n* = 250) of the drug indications were clearly stipulated (*p* < 0.05). Clear drug doses were stipulated on 52% (*n* = 89) of the CSCI prescriptions at baseline; post-intervention, 100% *(n* = 250) of the CSCI prescriptions contained clear drug doses (*p* < 0.05). The route of administration was stipulated on 58% (*n* = 99) of the CSCI prescriptions at baseline; post-intervention this increased to 100% (*n* = 250; *p* < 0.05).

Pre-intervention, 48% (*n* = 82) of the CSCI prescriptions stipulated a diluent, and of those specifying a diluent, only 23% (*n* = 40) stated a dilution volume. Post-intervention, all the CSCI prescriptions documented a diluent (*n* = 250; *p* < 0.05) and dilution volume (*n* = 250; *p* < 0.05). At baseline, 90% (*n* = 154) of the CSCI prescriptions directed an infusion duration; post-intervention, this increased to 100% (*n* = 250; *p* < 0.05).

### 3.2. Dose Ranges

The numbers of CSCI prescriptions that specified dose ranges were similar at baseline and post-intervention, at 71% (*n* = 122) and 72% (*n* = 179), respectively. At baseline, 69% (*n* = 84) of the CSCI prescriptions specifying a dose range were deemed safe; post-intervention, 97% (*n* = 173) were determined to be safe.

### 3.3. Mixing Directions

At baseline, mixing directions were not specified correctly on any CSCI prescriptions. Post-intervention, such directions were correct on 75% (*n* = 157; *p* < 0.05) of the prescriptions.

## 4. Discussion

The intervention was associated with a significant reduction in errors of omission, with prescriptions clearly stating drug indication, route of administration, drug dose, and infusion duration. This is important, as errors of omission can lead to patient harm, and CSCIs in the palliative demographic are associated with additional risks given the frequent use of high-risk, off-label medicines [14,15,16,17,18].

Prescription omission errors have been shown to delay medicine administration, result in missed doses, and lead to medicines being administered incorrectly [13,15]. This is because nursing staff may not have sufficient information to administer the required drug safely. Significant patient harm can result from this. Even though there were omission errors in the pre-intervention group, all of the CSCIs under investigation were administered. In the intervention group, there were no instances of omission errors. This was because the electronic prescription could not be completed unless all the required fields were populated. Overall, the findings of this study are in line with the available evidence in that electronic prescription interventions are linked to a notable decline in omission errors [13,19,20,21,22,23].

The omission of infusion length also raises safety concerns. For the most part, syringe drivers are administered over 24 h, and many syringe driver devices are actually ‘locked’ to this infusion duration [1,16,24,25]. The 17 CSCI prescriptions that did not specify a duration at baseline were administered over 24 h. It is not known if the nursing staff ‘assumed’ this duration or verbally confirmed it with the prescriber; either way, no prescription amendments were documented. There are situations when the duration of a syringe driver may be amended. When volumes larger than the capacity of the syringe driver device are required over 24 h, a syringe driver may be administered over 12 h so that it can be given twice in 24 h [25,26,27]. Therefore, the length of an infusion must be specified on safety grounds to ensure patients receive the correct dose over 24 h. Post-intervention, every CSCI chart specified an infusion duration of 24 h. It can, therefore, be seen that errors of omission concerning this prescription requirement were eliminated; however, this may have occurred at the expense of introducing new errors of commission. The electronic CSCI charts automatically defaulted to a duration of 24 h; therefore, prescribers may have failed to amend the infusion duration to 12 h where necessary. Unfortunately, the investigator did not review the clinical notes and management plans for every patient. Thus, the data collection did not identify such errors.

Failure to specify the route of administration may also lead to potential harm. In at least one of the cases where ‘route of administration’ was omitted, the syringe driver was administered as an intravenous infusion. Although CSCIs and intravenous infusions are widely considered equipotent, the latter requires the placement of an intravenous cannula, which is known to require more frequent replacing than its subcutaneous counterpart and is, overall, more invasive [28,29]. For the most part, syringe pumps should be administered by the subcutaneous route [1,16]. It is not known if the route of administration was ‘assumed’ to be intravenous or if it was verbally clarified. Interestingly, syringe drivers may be administered via the intravenous route if the patient has a central line in situ to allow reliable intravenous access. Additionally, if patients have significant hypotension (e.g., secondary to sepsis), then the intravenous route provides a more reliable route of drug administration, as subcutaneous absorption may be compromised secondary to reduced capillary perfusion [30]. Either way, omitting the route of administration may have led the patient to be unnecessarily subjected to the placement of an intravenous cannula. The electronic prescribing of CSCIs ensured a route of administration was documented for all prescriptions.

Total volume and diluent were frequently omitted from prescriptions at baseline. Such omissions can lead to medicines being mixed with the wrong diluent or being diluted to the incorrect volume, both of which may lead to physicochemical incompatibility [1,16]. The Department of Health’s legislation states that mixing medications in a syringe driver is permitted, provided the prescriber documents the instructions in writing and specifies exactly which components are to be mixed and in what ratios [31]. Additionally, the prescriber must determine if the individual components can be mixed together safely (i.e., they must be compatible). Due to the possibility that compatibility may vary according to concentration, the required diluent and total volume must be specified [1,16,31]. The prescriptions that had the required diluent or total volume omitted were still administered to patients, despite being of questionable legality. Consequently, the compatibility of these CSCIs could not be determined, and the nurses setting up the CSCIs likely selected the diluent. It is not known if nurse-led diluent selection is more accurate from a compatibility perspective when compared to doctor-led selection, although the former is outside of current legislation. Such issues appeared to be eliminated in the electronic prescribing group.

Failure to specify a therapeutic indication can lead to suboptimal symptom management, for example, only permitting the use of an opioid for pain management rather than pain and breathlessness [10]. It would appear the electronic intervention eliminated this issue and ensured all the CSCI prescriptions had a therapeutic indication stated.

For the prescriptions at baseline not specifying a dose, it was clear from the care records that there were delays in administration whilst the nurse involved spent time contacting the prescriber to clarify the intended dose. It would appear that the intervention successfully ensured all the prescriptions had a dose clearly stated, which as shown by Hindmarsh and Holden [13], had the potential to save significant nursing resources, as time did not need to be spent clarifying the prescribers’ intentions.

A large review [32] showed that one of the most common causes of medication errors is illegible handwriting. Illegible handwriting can lead to the misinterpretation of prescription directions and may result in serious mistakes (for instance, a ×10 overdose). However, potential harm from ambiguous handwriting was eliminated by the implementation of the intervention, as handwritten drug charts were no longer required.

The frequency at which dose ranges were prescribed remained unchanged pre- and post-intervention, with dose ranges appearing on a similar number of CSCI prescriptions across both study groups. The proportion of CSCI prescriptions specifying a safe dose range significantly improved in the intervention group. When populating CSCIs on EMIS, prescribers were provided with alerts to reinforce safe practice. It would, therefore, appear that the new intervention had the ability to improve compliance with local recommendations relating to dose ranges.

At baseline, of those syringe drivers requiring mixing directions, none were correctly stated (subdivisions of this group showed 25% contained incorrect mixing directions whilst, in the remaining 75%, directions were missing). This led to patients having the burden of multiple syringe drivers, each containing a single drug, which could have been mixed together in a single syringe. Post-intervention, of the syringe drivers requiring mixing directions, 75% contained legal and correct mixing directions. Of the remainder, 1% contained incorrect directions and, in the remaining 24% of cases, directions were missing. Overall, there was an improvement, which likely related to the electronic drug chart containing pre-populated mixing directions that the prescriber was able to select from.

## 5. Conclusions

The study demonstrated that the EMIS drug chart eliminated errors of omission, leading to potential improvements in both patient safety and service efficiency. It also showed for the first time that electronic systems can be used to ensure the safety of dose ranges and improve compliance with legislation surrounding the mixing of multicomponent infusions. Further research that has the sensitivity to identify all conceivable errors of commission is required. This is imperative, as the majority of investigations relating to the use of electronic prescribing systems in NHS Trusts have primarily focused on reductions in overall error rates, with limited attention given to new errors that may be generated.

Overall, the findings of this study are limited by the reliance upon short-term data and the lack of a control group. The outcomes are, thus, prone to confounding from various sources. Therefore, although the use of an EMIS-based drug chart appeared to be associated with improvements, this was not conclusive. Further evaluation using a controlled study design is warranted.

## Figures and Tables

**Table 1:** Prescribing errors at baseline and post-intervention.

Outcome Assessed	Baseline *N* = 171 (%)	Post-Intervention *N* = 250 (%)	*Significance*
**Errors of Omission**			
Drug stated	171 (100)	250 (100)	-
Indication stated	110 (64)	250 (100)	*p < 0.05*
Route stated	99(58)	250 (100)	*p < 0.05*
Clear dose stated	89 (52)	250 (100)	*p < 0.05*
Duration	154 (90)	250 (100)	*p < 0.05*
Signed and dated	171 (100)	250 (100)	*-*
Appropriate diluent specified	82 (48)	250 (100)	*p < 0.05*
Appropriate dilution volume	40 (23)	250 (100)	*p < 0.05*
**Dose ranges**			
Prescriptions stating a dose range	122 (71)	179 (72)	*-*
Safe dose ranges	84 (69)	173 (97)	*p < 0.05*
**Mixing directions**			
*N* of prescriptions requiring mixing directions	154 (90)	209 (84)	-
Correct mixing directions	0 (0)	157 (75)	
Incorrect mixing directions	56 (25)	1 (0.5)	*p* < 0.05
Mixing directions missing	98 (75)	51 (24)	

## Data Availability

There are no supporting or additional data for this article.
